# Adjuvant therapy for stage II melanoma: the need for further studies

**DOI:** 10.1016/j.ejca.2023.05.003

**Published:** 2023-05-08

**Authors:** Rebecca Lee, Mario Mandala, Georgina V. Long, Alexander M.M. Eggermont, Alexander C.J. van Akkooi, Shahneen Sandhu, Claus Garbe, Paul Lorigan

**Affiliations:** a The Christie NHS Foundation Trust, Department of Medical Oncology, Manchester, UK; b The University of Manchester, Division of Cancer Sciences, Manchester, UK; c University of Perugia, Perugia, Italy; d Melanoma Institute Australia, Faculty of Medicine and Health, The University of Sydney, Royal North Shore and Mater Hospitals, Sydney, Australia; e University Medical Center Utrecht & Princess Maxima Center, Utrecht, the Netherlands; f Comprehensive Cancer Center München, Technical University München & Ludwig Maximiliaan University, München, Germany; g Melanoma Institute Australia, Faculty of Medicine and Health, The University of Sydney, Royal Prince Alfred Hospital, Sydney, Australia; h Peter MacCallum Cancer Centre, The University of Melbourne, Melbourne, Australia; i Centre for Dermatooncology, Department of Dermatology, Eberhard Karls University, Tuebingen, Germany; j Ospedale Papa Givoanni XXIII, Bergamo, Italy

**Keywords:** Stage II melanoma, Adjuvant therapy, Clinical trials

## Abstract

Immunotherapy with checkpoint inhibitors has revolutionised the outcomes for melanoma patients. In the metastatic setting, patients treated with nivolumab and ipilimumab have an expected 5-year survival of > 50%. For patients with resected high-risk stage III disease, adjuvant pembrolizumab, nivolumab or dabrafenib and trametinib are associated with a significant improvement in both relapse-free survival (RFS) and distant metastasis-free survival (DMFS). More recently neoadjuvant immunotherapy has shown very promising outcomes in patients with clinically detectable nodal disease and is likely to become a new standard of care. For stage IIB/C disease, two pivotal adjuvant trials of pembrolizumab and nivolumab have also reported a significant improvement in both RFS and DMFS. However, the absolute benefit is low and there are concerns about the risk of severe toxicities as well as long-term morbidity from endocrine toxicity. Ongoing registration phase III trials are currently evaluating newer immunotherapy combinations and the role of BRAF/MEK-directed targeted therapy for stage II melanoma. However, our ability to personalise therapy based on molecular risk stratification has lagged behind the development of novel immune therapies. There is a critical need to evaluate the use of tissue and blood-based biomarkers, to better select patients that will recur and avoid unnecessary treatment for the majority of patients cured by surgery alone.

## Introduction

Immunotherapy with checkpoint inhibitors (CPI) and targeted therapy have significantly improved outcomes for patients with stage IV melanoma such that we can realistically discuss durable responses and even cure in a significant subset of patients. However, these therapies may cause acute and long-lasting toxicities. Moving these therapies to an earlier disease stage setting where a significant proportion of patients are cured with surgery alone changes the balance between risk and benefit and highlights the critical importance for biomarker development to allow molecular-based risk stratification.

## Stage III melanoma: adjuvant and neoadjuvant treatment

Adjuvant treatment for 12 months with either an anti-PD-1 inhibitor, (pembrolizumab, nivolumab) or BRAF and MEK inhibitors (dabrafenib plus trametinib for patients with an activating BRAFV600 mutation) are now established as a standard of care for resected stage III and stage IV melanoma, based on the results from three pivotal trials. These trials showed a consistent benefit in relapse-free survival (RFS) and distant metastasis-free survival (DMFS) for all three treatment approaches within the patient populations studied [1–7]. As yet, none of these pivotal trials have reported mature overall survival (OS) data, but have reported no significant difference in impact on overall Health-related Quality of Life (HRQoL) scales [8–11]. In the Check-mate 238 study, at 5-year follow-up, OS rates in the intention to treat population were 76% and 72%, for the nivolumab and ipilimumab arm, respectively; nevertheless, data were immature, since only 228 of the expected 302 mortality events had occurred [12].

More recently, a number of novel CPI combination approaches are under evaluation. Relatlimab is an inhibitor of the immune checkpoint LAG-3 which has shown significant activity in the metastatic setting [13]. A recent study (RELATIVITY 047) comparing nivolumab plus relatlimab versus single-agent nivolumab in 714 patients with unresectable stage III and stage IV showed a significant improvement in median progression-free survival (PFS) (10.2 [95% confidence interval {CI} 6.5–14.8] versus 4.6 [95% CI 3.5–6.4]) months, Hazzard Ratio (HR 0.78 [0.64–0.94]) [14,15]. There was a trend towards improvement in OS (HR 0.80 [0.64–1.01], p = 0.0593) for the combination arm with OS rates at 24 months 63.7% (95% CI, 58.1–68.7) with relatlimab plus nivolumab versus 58.3% (95% CI, 52.7–63.4) with nivolumab [14,15]. There was a modest increase in grade 3–4 toxicity (21.1% versus 11.1%) in the combination arm [15]. This combination is now being evaluated as adjuvant therapy in resected stage III disease in the Relativity-098 trial (NCT05418972). The combination of cemiplimab (anti-PD-1 MAb) and fianlimab (anti-LAG-3 MAb) is also being compared to pembrolizumab in patients with resected stage IIc–IV melanoma (NCT05608291). Vibostolimab is an anti–T-cell immunoreceptor antibody with Ig and ITIM domains (TIGIT) [16]. The combination of pembrolizumab and vibostolimab has recently shown activity as neoadjuvant therapy in stage III melanoma [17], and is now being compared to pembrolizumab in 1560 patients resected stage II–IV disease (KEYVIBE-010, NCT05665595). Based on positive results from Keynote-942, a randomised phase 2b study of pembrolizumab and the Moderna personalised mRNA-4157/V940 vaccine, this combination has recently been given breakthrough therapy designation for the management of resected high-risk melanoma [18].

Neoadjuvant treatment has also been shown to have significant activity in macroscopic stage III melanoma with clinically detectable nodes, and is set to become a standard of care [19]. The greater activity of neoadjuvant versus adjuvant immune therapy is likely due to the presence of a broad repertoire of tumour antigens at time of CPI administration, allowing for wider and deeper T-cell response [20]. Additionally, ascertainment of pathological responses to neoadjuvant CPI has been shown to correlate strongly with RFS, thereby allowing for tailoring of post-surgical management and surveillance [19]. In the SWOG S1801 phase 2 randomised trial (NCT03698019), 313 patients with resectable stage III–IV melanoma were randomised to receive either surgery followed by 18 cycles of pembrolizumab 200 mg (flat dose) every 3 weeks, or three cycles of pembrolizumab administered as neoadjuvant therapy, followed by an additional 15 cycles post-operatively [21]. With a median follow-up of 14 months, the 2-year estimated event-free survival was 72% in the neoadjuvant group and 49% in the adjuvant group (HR = 0.58, p = 0.004) [21]. The toxicity profile was similar in both arms and administration of neoadjuvant pembrolizumab was not associated with increased complications. As yet, the pathological response rate, and its correlation with longer-term efficacy outcomes in the neoadjuvant arm has not been reported. Other studies evaluating neoadjuvant combination ipilimumab + nivolumab have reported significant activity [20,22]. The OpACIN Study showed a major pathological response (MPR; ≤ 10% tumour cells remaining after neoadjuvant therapy) rate of 60% for neoadjuvant ipilimumab 3 mg/kg + nivolumab 1 mg/kg, but at the expense of significant toxicity with 90% of patients developing grade 3/4 adverse events [20]. The OpACIN-neo study examined the toxicity of three different combination regimens in the neoadjuvant setting in 86 patients, and showed a MPR rate of 64% and 20% grade 3 toxicity for ipilimumab 1 mg/kg and nivolumab 3 mg/kg for two cycles [22]. This regimen has been taken forward in the PRADO trial that examined the de-escalation of both surgery and systemic therapy in patients who achieve a MPR [23]. The phase 3 NADINA study [24], which is comparing neoadjuvant ipilimumab + nivolumab versus the standard adjuvant CPI in patients with resectable stage III disease (NCT04949113) is currently ongoing. Based on the PRADO data, de-escalation of adjuvant CPI in patients with MPR has been incorporated into the phase 3 NA-DINA trial design [24]. The combination of relatlimab and nivolumab (two neoadjuvant doses followed by surgery then ten doses of adjuvant combination therapy) has also shown early promise with a pathological complete response (CR) of 57% [25]. Other early phase studies including the multi-arm Morpheus study (NCT05116202) testing different combinations of neoadjuvant CPI are ongoing.

## Can stage III treatment approaches be used in stage II disease?

Outcomes for patients with stage II melanoma are heterogeneous, varying from patients with a low risk of death (stage IIA 94% survival at 10 years) to patients with stage IIC melanoma (10 years survival 75%), which has a worse prognosis than patients with stage IIIA disease (10 years survival 88%) and similar to patients with stage IIIB (10 years survival 77%) [26]. Of note, large studies conducted by the German Registry and the EORTC which include a mix of real-world data and patients in clinical trials, report a worse outcome across all stage I–III sub-groups compared to the AJCC report [27,28]. For stage II melanoma, whilst the individual risk is low or moderate, because earlier-stage melanoma is more commonly diagnosed than late-stage disease, these patients account for approximately 30–50% of all melanoma deaths [29]. As a result, both adjuvant pembrolizumab and adjuvant nivolumab have been evaluated in resected stage IIB/C melanoma. The Keynote 716 study (NCT03553836) compared adjuvant pembrolizumab 200 mg IV three weekly for 1 year to placebo in 976 patients with resected stage IIB and IIC melanoma [30]. With a median follow-up of 27.9 months, there was a significant improvement in both RFS (RFS HR 0.64 [0.50–0.84]) and DMFS 0.64 (0.47–0.88), p = 0.003, for pembrolizumab compared to placebo, resulting in approval for this indication by both the U.S. Food and Drug Administration (FDA) and European Medicines Agency (EMA) [30]. HRQoL outcomes showed no meaningful difference between treatment arms [31]. More recently, the primary analysis of the CheckMate 76K study has shown a similar outcome for adjuvant nivolumab in this patient population [32]. This study randomised 790 patients with resected stage IIB/C melanoma 2:1 to with either nivolumab 480 mg IV 4-weekly for 1 year, or to placebo. With a median follow-up of 15.8 months, the RFS at 1 year was 89% for the nivolumab arm and 79% in the control arm (HR 0.42, p < 0.001) and 1-year DMFS was 92% in the nivolumab arm and 87% in the control arm, (HR 0.47 [0.30–0.72]) [32]. These studies are a major step forward and great news for patients, providing additional treatment options.

## Other trials in stage II melanoma

Where next from here? Based on the activity of adjuvant dabrafenib plus trametinib in resected BRAF mutant stage III disease, where 5-year follow-up results showed an ongoing significant impact on both RFS and DMFS, BRAF-directed therapy is under evaluation in patients with resected stage II disease. The EORTC-2139/COL-UMBUS AD study (NCT05270044) is randomising 815 patients with resected stage IIB and IIC BRAFV600 mutated melanoma to either 12 months of adjuvant therapy with encorafenib plus binimetinib or with placebo. Other studies are looking at combination immunotherapy. The KEYVIBE-101 study outlined above also includes patients with resected stage IIB and IIC disease, and the study with cemiplimab + fianlimab is including patients with resected stage IIc disease. Neoadjuvant approaches are also being examined. There are two phase II trials in progress to test feasibility, including NeoReNi II (NCT05418972) which is investigating combination nivolumab-relatlimab and another trial (NCT03757689) investigating single-agent pembrolizumab.

## Potential drawbacks of adjuvant therapy in stage II disease

A fundamental question is whether we should treat all eligible patients with resected AJCC stage IIB/C melanoma patients with adjuvant immunotherapy. A recent study carried out in the United States looked at the decisions made by eligible patients being offered adjuvant pembrolizumab for resected stage IIIB/C/IV melanoma reported a 14/34 patients (41%) opted for adjuvant anti-PD1 immunotherapy, 20/34 (59%) opted for observation [33]. The benefits of adjuvant pembrolizumab in stage II disease are modest, with a 5.9% absolute reduction in the risk of developing distant metastases at 2 years [30]. Similar findings were seen for nivolumab with a 5% difference in DMFS at 12 months [32], an end-point that is often taken as a surrogate for OS. Many patients are not suitable for immunotherapy due to co-morbidities, or are concerned about potential toxicity. Grade 3–4 treatment-related adverse events were reported in 17% of the pembrolizumab patients in Keynote 716% and 10% of nivolumab patients in CheckMate76K [30,32]. Long-term endocrine toxicity was common, with 17–19% developing thyroiditis. Potentially life-limiting endocrine toxicity occurred in a number of patients including hypophysitis (3%), adrenal insufficiency (3%), insulin-dependent diabetes (< 1%) and there was one treatment-related death with nivolumab [30,32]. Emerging data in patients treated with immunotherapy in both the adjuvant and metastatic settings suggest an increased risk of other serious long-term toxicities, including an increased risk of atherosclerotic cardiovascular events in those with risk factors for these [34–37]. Furthermore, there are unanswered questions about the potential impact of prior adjuvant immunotherapy if patients go on to develop metastatic disease. A number of retrospective analyses (including small numbers of patients progressing on adjuvant therapy) have shown that following progression on anti-PD-1 therapy, the response rate to anti-CTLA-4 monotherapy is ~10–15% and ~30% for anti-CTLA-4 combined anti-PD-1, which is significantly less than in immune therapy naïve patients [38–40]. Data from the EORTC 1325/Keynote 054 study showed that in patients previously treated with pembrolizumab who relapsed > 6 months after receiving it and were rechallenged with the same treatment, the response rate was only 15% and median PFS was 4.1 months (95% CI 2.6-NE) [41]. It remains unclear whether these outcomes reflect the selection of a population of patients that were intrinsically resistant to CPI, or whether the prior exposure promoted a resistant phenotype. Data from both the DREAMseq and the SECOMBIT studies confirm previous observations that for patients with metastatic disease harbouring a BRAF V600 mutation, the best chance of long-term survival is with first-line immunotherapy [42–44]. Elegant pre-clinical data have shown that resistance to targeted therapy drives cross-resistance to immune therapy through resistant cells creating an immunosuppressive tumour microenvironment that lacks functional CD103^+^ dendritic cells, precluding an effective T cell response [45]. A better understanding of the effect of prior adjuvant therapy on likelihood of response when patients have progressed is needed.

## How can we improve patient selection?

A different strategy is to utilise emerging blood and tissue biomarkers to better define risk groups, enriching for those at highest risk of recurrence who would benefit most from adjuvant strategies, or potentially allowing escalation and de-escalation of treatment in an individualised risk approach based on real-time biomarker assessment. A number of tissue-based gene expression scores have been developed including MelaGenix, Skyline, Decision-Dx melanoma, and CAM-121 [46–50]. The NivoMela study (NCT04309409) is utilising the eight gene MelaGenix GEP-based score to identify patients at high risk for relapse [46]. Approximately 60% of patients with stage II melanoma will be classified as high risk and randomised 2:1 to receive either nivolumab as adjuvant treatment or observation. The 40% of patients in the low-risk category will not receive adjuvant therapy. One of the issues with tissue-based biomarkers are their low positive predictive value [46]. However, this strategy is better at excluding patients who are lower risk [46], for example, the CP-GEP algorithm low-risk patients had a 5 years RFS of 93% [47]. Potentially they can therefore reduce the amount of unnecessary adjuvant treatment offered to some patients.

Circulating tumour DNA (ctDNA) is emerging as a powerful tool for the detection of minimal residual disease and is being examined across a number of tumour types (including breast [51], colorectal [52,53], bladder [54], melanoma [55]). Previous work from our groups and others have shown that the detection of ctDNA after resection of stage II or III disease is highly specific for an increased rate of recurrence and poorer OS [56–58]. Although sensitivity of the assays immediately following surgery are an issue, this can be improved through longitudinal sampling [57]. The DETECTION Study (NCT04901988) is monitoring patients with resected stage IIB/C/IIIA melanoma using clinical review, imaging, and regular blood tests for ctDNA. Patients will be randomised to either adjuvant therapy for 12 months, or to follow-up with ctDNA. Patients in the experimental arm experiencing a local recurrence will have surgery followed by adjuvant therapy as standard of care with ongoing ctDNA monitoring. Those with detectable mutations in ctDNA will be offered systemic therapy according to clinicians’ choice, most likely immunotherapy. The primary end-point is DMFS. The trial will assess whether a ctDNA-guided approach could prevent distant metastases whilst sparing the approximately 75–80% of patients who are cured by surgery alone from the unnecessary cost and potential toxicity of adjuvant treatment.

## Why we need more data?

Completion of the EORTC-2139/COLUMBUS-AD, NivoMela, and DETECTION studies is essential to better inform how to maximise treatment options, sequence treatment, reduce risk for chronic toxicity, enrich for patients who need treatment and not treat those who will derive no benefit. In addition, studies examining whether duration of CPI treatment could be reduced, for example, 6 months instead of 12 months, would potentially reduce the time burden for patients and cost to healthcare systems. Immunotherapy has brought huge benefits to patients with melanoma, but it is unlikely that this is the right approach for all lower-risk patients after surgery. The magnitude of the benefit is modest, the risk of toxicity real, and the costs in terms of both drug acquisition and resource utilisation are high. Once we can identify the subset of patients who will recur, we can focus on selecting the best treatment, improving the outcomes and reducing the risks for this patient population. In addition, identifying those patients not requiring treatment will have huge benefits but brings extra challenges. Conducting equivalence studies using de-escalation approaches relies on academic groups and this is usually not aligned with the strategy of the Pharmaceutical Industry. Without these studies, we risk limiting treatment options to immediate immunotherapy for all, with the only question being whether newer combinations are better.
